# l-Theanine Ameliorates d-Galactose-Induced Brain Damage in Rats via Inhibiting AGE Formation and Regulating Sirtuin1 and BDNF Signaling Pathways

**DOI:** 10.1155/2021/8850112

**Published:** 2021-07-19

**Authors:** Li Zeng, Ling Lin, Ling Chen, Wenjun Xiao, Zhihua Gong

**Affiliations:** ^1^Key Lab of Tea Science of Ministry of Education, Hunan Agricultural University, Changsha, Hunan 410128, China; ^2^School of Pharmacy, Shaoyang University, Shaoyang 422000, China; ^3^National Research Center of Engineering and Technology for Utilization of Botanical Functional Ingredients, Hunan Agricultural University, Changsha 410128, China

## Abstract

The maintenance of homeostasis is essential for mitigating stress and delaying degenerative diseases such as Alzheimer's disease (AD). AD is generally defined as the abnormal production of *β*-amyloid (A*β*) and advanced glycation end products (AGEs). The effects of l-theanine on A*β* and AGE generation were investigated in this study. Decreased AGEs and A*β*_1-42_ levels were reflected by increased acetylcholine (ACh) concentration and acetylcholinesterase **(**AChE**)** activity inhibition compared to model rats. l-Theanine also inhibited nuclear factor-*κ*B (p65) protein expression by activating sirtuin1 (SIRT1), reducing inflammatory factor expression, and downregulating the mRNA and protein expression of AGE receptors (RAGE). Superoxide dismutase 2 and catalase protein expressions were markedly upregulated by l-theanine, whereas oxidative stress-related injury was alleviated. The expression of peroxisome proliferator-activated receptor-*γ* coactivator 1*α* (PGC-1*α*) was also found to be increased. H&E staining showed that the apoptosis of hippocampal neurons was mitigated by decreased Bax and cleaved-caspase-3 protein expression and the increase of Bcl-2 protein expression. Moreover, l-theanine increased the gene and protein expression of brain-derived neurotrophic factor (BDNF). These findings suggest that the potential preventive effects of l-theanine against AD may be attributed to its regulation of SIRT1 and BDNF proteins and its mitigation of AGEs/RAGE signaling pathways in the brain tissue of AD model rats.

## 1. Introduction

Advanced glycation end products (AGEs) accumulate in human and animal serum or tissues with age [1, 2]. Clinical studies have found that the amount of AGE-modified *β*-amyloid protein (A*β*) in the cerebrospinal fluid of patients with Alzheimer's disease (AD) is three times higher than that in healthy individuals [3]. AD is a progressive neurodegenerative disease associated with abnormal deposition of A*β* in the brain [4]. Under normal conditions, the production and clearance of A*β* in the body are in dynamic equilibrium. When A*β* metabolism is dysfunctional, this balance is disrupted, and A*β* accumulates, resulting in neurotoxicity [5]. Oxidative stress, mitochondrial dysfunction, inflammation, and neuronal cell apoptosis are closely related to AD pathogenesis [6]. Receptor for AGEs (RAGE), a multiligand membrane receptor primarily expressed in neurons and immune cells [7], binds AGEs and A*β*, thus activating various signaling pathways in brain tissues, resulting in oxidative damage, mitochondrial dysfunction, chronic inflammation, protein degeneration, and apoptosis [7–9]. These mechanisms can disrupt brain cell homeostasis, ultimately leading to a degeneration of the normal brain structure and function [9, 10].

Since excess d-galactose exceeds the body glucose metabolism capacity, the Maillard reaction can occur between the aldehyde and protein free amino groups, resulting in the formation of AGEs [11, 12]. In addition, in the presence of oxidative stress, glucose produced by d-galactose metabolism is oxidized to produce active carbonyl compounds (such as methylacetaldehyde), which also promote the formation of AGEs [1]. Studies have shown that the brain tissue is unable to metabolize excess d-galactose and that the gradual decrease in brain function induced by d-galactose positively correlates with AGE formation, A*β* deposition, decrease in acetylcholine (ACh) levels, neuronal apoptosis, and loss of brain-derived neurotrophic factor (BDNF) in brain tissues [13]. Sirtuin1 (SIRT1) is an important functional protein in various mammalian metabolic tissues and can regulate the biological processes involved in neurodegenerative diseases, such as inflammation, oxidative stress, mitochondrial function, cell senescence, and apoptosis [14]. Sirtuin1 elicits its effects by activating downstream transcription factors and has shown the potential to attenuate d-galactose-induced brain aging in rats [15, 16]. BDNF is a key factor that promotes neuronal survival and development and improves learning and memory [17, 18]. Additionally, SIRT1 and BDNF may be key factors in AGE production and cognitive impairment in d-galactose-induced glycosylation model rats [19, 20].

Food nutrition intervention aimed at inhibiting the production of AGEs, reducing the accumulation of A*β* in brain tissue, downregulating the expression of RAGE protein, and maintaining homeostasis in brain tissue may be an effective approach to treat and prevent AD [7, 21]. Epidemiological studies have shown that daily tea drinking can reduce the risk of neurodegenerative diseases, such as cognitive decline and cognitive impairment in the elderly [22, 23]. l-Theanine from tea can increase the amount of glutathione (GSH) and ACh and the activities of antioxidant enzymes, such as superoxide dismutase (SOD), catalase (CAT), and GSH-peroxidase (Px); promote the synthesis and secretion of BDNF; decrease the amount of malondialdehyde (MDA); and maintain a balance expression of inflammatory factors in brain tissue, thereby reducing mitochondrial damage induced by external stress, such as cadmium, A*β*_1-42_, and polychlorinated biphenyls (e.g., Aroclor1254), and inhibiting apoptosis in brain neuronal cells [23, 24]. At the same time, *in vitro* studies have shown that l-theanine reduces AGE formation [25]. We have previously reported that l-theanine can protect the liver, promote AGE metabolism, and reduce the amount of AGEs in the liver and serum by maintaining the balance between oxidative stress and inflammation in the liver tissues of d-galactose-induced model rats [26, 27]. However, d-galactose can circulate in the blood, therefore reaching various tissues and organs and form AGEs [1, 8]. To the best of our knowledge, no reports have described the effects of l-theanine on AGE formation in brain tissues.

Therefore, d-galactose-induced glycosylated model rats were used to explore the mechanisms underlying l-theanine maintenance of homeostasis in brain tissues in this study. We further investigated the role of l-theanine in mitigating the effects of adverse factors, such as brain inflammation, oxidative stress, mitochondrial function, cell senescence, and apoptosis mediated by AGEs/RAGE. Our findings may provide a scientific basis for the development of functional products based on l-theanine to promote healthy aging.

## 2. Materials and Methods

### 2.1. Materials and Reagents

Healthy male Sprague-Dawley rats (6 weeks old, specific pathogen-free, weighing 180–200 g) were obtained from Hunan Slack Jingda Experimental Animal Co., Ltd. (China) (animal quality certificate number: 43004700043916). l-Theanine (cat. no. G5388) and d-galactose (cat. no. SMB00395; purity ≥ 99%) were purchased from Sigma (St. Louis, MO, USA). Enzyme-linked immunosorbent assay (ELISA) kits for AGEs (cat. no. CSB-E09413r), A*β*_1-42_ (cat. no. CSB-E10786r), tumor necrosis factor- (TNF-) *α* (cat. no. CSB-E11987r), interleukin- (IL-) 1*β* (cat. no. CSB-E08055r), IL-6 (cat. no. CSB-E04640r), and neuronal nitric oxide synthase (nNOS; cat. no. CSB-E14034r) were purchased from Wuhan Huamei Biological Engineering Co., Ltd. (China). Kits for evaluation of the expression or activity of SOD (cat. no. A001-3), CAT (cat. no. A007-1), GSH-Px (cat. no. A005), NOS (cat. no. A014-2), total antioxidant capacity (T-AOC; cat. no. A015-2), MDA (cat. no. A003-1), ACh (cat. no. A105-1), and acetylcholinesterase (AChE; cat. no. A024-1-1) were purchased from Nanjing Jiancheng Institute of Bioengineering (China). Antibodies targeting *β*-actin (cat. no. 60008-1--Ig), RAGE (cat. no. 16346-1-AP), SOD2 (cat. no. 24127-1-AP), CAT (cat. no. 14195-1-AP), SIRT1 (cat. no. 13161-1-AP), peroxisome proliferator-activated receptor-*γ* coactivator- (PGC-) 1*α* (cat. no. 66369-1-Ig), nuclear factor- (NF-) *κ*B (p65) (cat. no. 10745-1-AP), Bcl-2 (cat. no. 26593-1-AP), Bax (cat. no. 50599-2-Ig), cleaved-caspase-3 (cat. no. 19677-1-AP), and BDNF (cat. no. 66292-1-Ig), as well as horseradish peroxidase (HRP) goat anti-mouse IgG (cat. no. SA00001-1) and HRP goat anti-rabbit IgG (cat. no. SA00001-2) were purchased from Proteintech Company (USA). Anti-Ace-NF-*κ*B antibodies (p65; cat. no. ab19870) were purchased from Abcam (Cambridge, UK).

### 2.2. Experimental Animal Design

Rats were housed in separate cages in a room with a constant temperature (25 ± 2°C) and humidity (50–60%), with a light cycle set to 14 h of light/10 h of darkness. Before the experiment, all 30 rats were provided with standard rat chow and water ad libitum. After 1 week for acclimation, the rats were divided into five groups (*n* = 6 rats/group) according to different treatment methods, as follows: (1) control group (CON), rats were fed the corresponding dose of normal saline according to their body weight, and the corresponding volume of normal saline was injected into the back of the neck; (2) model group (MOD), rats were fed the corresponding dose of physiological saline according to body weight and were injected with 200 mg/kg body weight (BW) of d-galactose physiological saline solution subcutaneously into the back of the neck; (3) low-dose l-theanine group (L-LT), rats were fed 100 mg/kg BW of l-theanine physiological saline solution and were injected with 200 mg/kg BW of d-galactose subcutaneously into the back of the neck; (4) medium-dose l-theanine group (M-LT), rats were fed 200 mg/kg BW of l-theanine physiological saline solution and simultaneously injected with 200 mg/kg BW of d-galactose into the back of the neck; and (5) high-dose l-theanine group (H-LT), rats were fed 400 mg/kg BW of l-theanine physiological saline solution and injected with 200 mg/kg BW of d-galactose subcutaneously into the back of the neck.

The rats were treated once a day for 56 days. After 8 weeks, all rats fasted but were allowed free access to water *ad libitum*. Rats were anesthetized by intraperitoneal injection of 3% pentobarbital sodium, and rat brain tissues were harvested and stored in a cryogenic refrigerator at -80°C (Thermo Corporation, USA). The experimental animal program was approved by the Animal Experiment Ethics Committee of Hunan Agricultural University (No: 015063506, Changsha, China) and was strictly implemented in accordance with the 8th edition of the US Guidelines for the Care and Use of Laboratory Animals.

### 2.3. Sample Analysis

#### 2.3.1. Histopathological Detection and Analysis of Hippocampal Tissue in Experimental Rats

With reference to the methods reported by Zeng et al. [26], the same part of the hippocampal tissue from three rats in each group was embedded in paraffin and stained with hematoxylin-eosin (H&E), and pathological changes in the hippocampal tissue were observed under a 400x light microscope (Leica Microsystems AG, Wetzlar, Germany).

#### 2.3.2. Detection of AGEs and A*β*_1-42_ in the Brain Tissues of Experimental Rats Using ELISA

Approximately 0.02 g tissue was collected from the same part of the brain in each rat, and the amount of AGEs and A*β*_1-42_ in the brain tissue was detected according to the manufacturer's instructions.

#### 2.3.3. Biochemical Detection of ACh and AChE in Experimental Rat Brain Tissue

Approximately 0.02 g tissue was collected from the same part of the brain in each rat, and the contents of ACh and AChE in the brain tissues were detected using an enzyme labeling instrument according to the manufacturer's instructions.

#### 2.3.4. Detection of Oxidative Stress in the Brain Tissues of Experimental Rats

Approximately 0.02 g tissue was collected from the same part of the brain in each rat. The levels of SOD, CAT, GSH-Px, NOS, T-AOC, and MDA in the brain tissues of each group were detected using an enzyme labeling instrument according to the instructions provided by the kit manufacturer.

#### 2.3.5. Detection of Inflammatory Cytokines in the Brain Tissues of Experimental Rats Using ELISA

Approximately 0.02 g tissue was collected from the same part of the brain in each rat. According to the manufacturer's instructions, the protein contents of TNF-*α*, IL-1*β*, IL-6, and nNOS were measured using an enzyme labeling instrument.

#### 2.3.6. Real-Time Quantitative Polymerase Chain Reaction (qPCR)

The mRNA expression levels of *RAGE*, *SIRT1*, *PGC-1α*, and *BDNF* in brain tissues were measured using qPCR. Approximately 0.05 g tissue was collected from the same part of the brain in each rat, and total RNA was extracted using TRIzol reagent (Invitrogen, Carlsbad, CA, USA). The RNA was then reverse transcribed into cDNA, and an ABIQuantStudio3 fluorescence qPCR instrument was used for PCR amplification. The PCR cycle parameters were as follows: predenaturation at 95°C for 10 min, followed by 40 cycles of denaturation at 95°C for 15 s, and annealing at 60°C for 60 s. The 2^–△△*Ct*^ method was used to calculate the expression level of each gene in each sample relative to that in the control group. The sequences of the target genes were obtained from NCBI, and primers were designed using PrimerPremier5. All primers were synthesized by Shanghai Shenggong Biological Engineering Co., Ltd. (Table 1).

#### 2.3.7. Western Blotting

With reference to the methods reported by Zeng et al. [26], homogenates from 0.05 g hippocampal tissue from three rats in each group were randomly selected, and protein was extracted after cleavage. Protein concentrations were then determined. Samples were boiled and denatured, and proteins were separated by electrophoresis and transferred to membranes. Membranes were then incubated with appropriate primary antibodies, washed, and incubated with secondary antibodies. Protein bands were detected using enhanced chemiluminescence. The molecular weight and optical density of target bands for RAGE, SOD2, CAT, SIRT1, PGC-1*α*, NF-*κ*B (p65), Ace-NF-*κ*B (p65), BDNF, Bcl-2, Bax, and cleaved-caspase-3 were analyzed using a gel image processing system (Media Cybernetics, Inc., Rockville, MD, USA).

### 2.4. Statistical Analysis

The data were processed using the GraphPadPrism6 software (GraphPad Software, Inc., USA), and the results are expressed as means ± standard deviations. Single-factor analysis of variance and least significant difference tests were used to compare the significance of differences between groups. Differences with *P* values <0.05 were considered significant.

## 3. Results

### 3.1. Pathological Changes in the Hippocampal Tissues of Rats in Each Group

**Figure 1:**
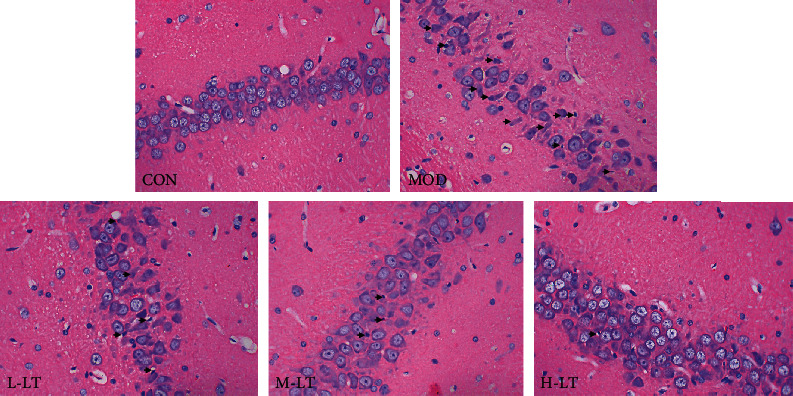
Pathological changes in rats' hippocampal tissues of each group (400x; *n* = 3). The black arrows indicate shrinkage or apoptosis of nerve cells. L-LT: low-dose (100 mg/kg) l-theanine group; M-LT: medium-dose (200 mg/kg) l-theanine group; H-LT: high-dose (400 mg/kg) l-theanine group.

Hippocampal neurons in the MOD group, which were induced by d-galactose, were arranged in a more disordered manner and showed higher shrinkage or apoptosis (black arrow) in comparison with the CON group (Figure 1). With the l-theanine treatment, the damage was substantially reduced, resulting in a neat arrangement of the cells. The effects obtained with the highest concentration of l-theanine were similar to the CON group, which indicate a potential protective effect of l-theanine in the hippocampal tissues.

### 3.2. Effects of l-Theanine on AGEs and A*β*_1-42_ in the Brain Tissues of Experimental Rats

**Figure 2:**
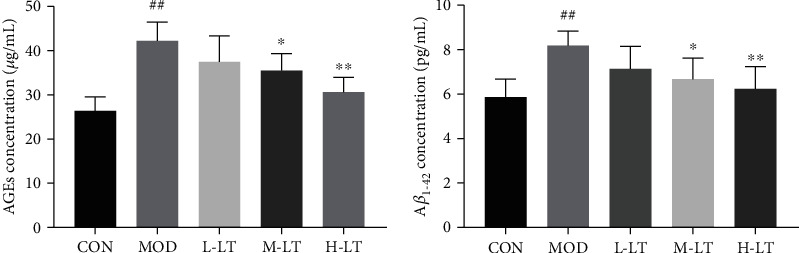
AGEs and A*β*_1-42_ concentrations in rats' brain tissues of each group (*n* = 6). Compared with the CON group: ^#^*P* < 0.05, ^##^*P* < 0.01; compared with the MOD group: ^∗^*P* < 0.05, ^∗∗^*P* < 0.01. L-LT: low-dose (100 mg/kg) l-theanine group; M-LT: medium-dose (200 mg/kg) l-theanine group; H-LT: high-dose (400 mg/kg) l-theanine group.

As shown in Figure 2, the concentrations of AGEs and A*β*_1-42_ in the brain tissues of the MOD group were significantly (*P* < 0.01) higher than those of the CON group. Additionally, following the administration of medium (200 mg/kg) and high-dose (400 mg/kg) of l-theanine, the concentration of AGEs and A*β*_1-42_ in the brain tissues significantly decreased in a dose-dependent manner. However, there was no significant decrease in the L-LT group.

### 3.3. Effects of l-Theanine on ACh and AChE in the Brain Tissues of Experimental Rats

**Figure 3:**
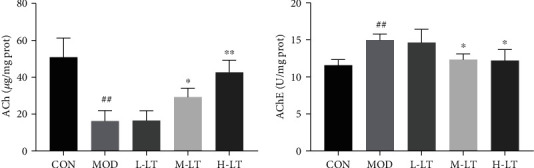
The concentration of ACh and AChE in rats' brain tissues of each group (*n* = 6). Compared with the CON group: ^#^*P* < 0.05, ^##^*P* < 0.01; compared with the MOD group: ^∗^*P* < 0.05, ^∗∗^*P* < 0.01. L-LT: low-dose (100 mg/kg) l-theanine group; M-LT: medium-dose (200 mg/kg) l-theanine group; H-LT: high-dose (400 mg/kg) l-theanine group.

The amounts of ACh and AChE in the brain tissues are shown in Figure 3. Compared with the CON group, the amounts of ACh and AChE were significantly decreased and increased, respectively, in the brain tissues of the MOD group (*P* < 0.01). Following l-theanine treatment, the level of ACh and AChE in brain tissues significantly improved and decreased, respectively, in a dose-dependent manner (*P* < 0.05 and *P* < 0.01, respectively).

### 3.4. Effects of l-Theanine on Oxidative Stress in Brain Tissues of Experimental Rats

**Figure 4:**
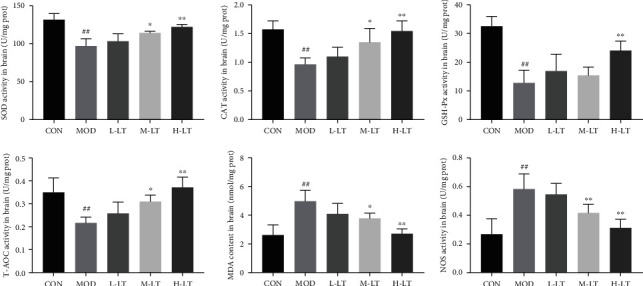
Activities and concentrations of oxidative stress-associated markers in rats' brain tissues of each group (*n* = 6). Compared with the CON group: ^#^*P* < 0.05, ^##^*P* < 0.01; compared with the MOD group: ^∗^*P* < 0.05, ^∗∗^*P* < 0.01. L-LT: low-dose (100 mg/kg) l-theanine group; M-LT: medium-dose (200 mg/kg) l-theanine group; H-LT: high-dose (400 mg/kg) l-theanine group.

Evaluation of oxidative stress response to l-theanine is shown in Figure 4. SOD, CAT, GSH-Px, and T-AOC activities were significantly decreased, whereas NOS activity and MDA concentration were significantly increased in brain tissues after d-galactose exposure (*P* < 0.01). After administration of medium and high-dose of l-theanine, SOD, CAT, and T-AOC activities were significantly increased, whereas NOS activity and MDA concentration significantly decreased (*P* < 0.05 and *P* < 0.01). Similarly, there were no significant changes in oxidative stress levels in brain tissues treated with a low concentration of l-theanine (*P* > 0.05).

### 3.5. Effects of l-Theanine on Inflammatory Cytokines in Brain Tissues from Experimental Rats

**Figure 5:**
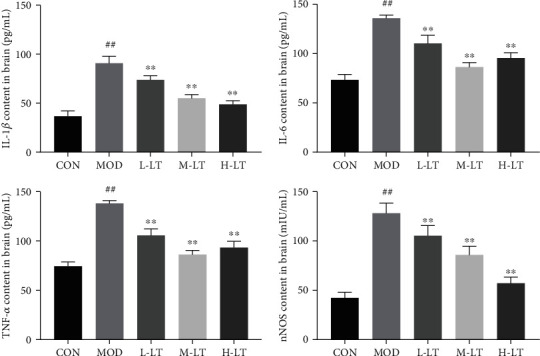
Concentrations of IL-1*β*, IL-6, TNF-*α*, and nNOS in rats' brain tissues of each group (*n* = 6). Compared with the CON group: ^#^*P* < 0.05, ^##^*P* < 0.01; compared with the MOD group: ^∗^*P* < 0.05, ^∗∗^*P* < 0.01. L-LT: low-dose (100 mg/kg) l-theanine group; M-LT: medium-dose (200 mg/kg) l-theanine group; H-LT: high-dose (400 mg/kg) l-theanine group.

Inflammatory cytokines play important roles in age-related degenerative diseases [28]. As shown in Figure 5, inflammatory factors, including IL-1*β*, IL-6, TNF-*α*, and nNOS, in the brain tissues of the MOD group were significantly higher than those of the CON group (*P* < 0.01). Compared with the MOD group, the levels of IL-1*β*, IL-6, TNF-*α*, and nNOS in the brain tissues of the l-theanine-treated groups were significantly decreased (*P* < 0.01).

### 3.6. Effects of l-Theanine on RAGE, SIRT1, PGC-1*α*, and BDNF mRNA Levels in the Brains of Experimental Rats

**Figure 6:**
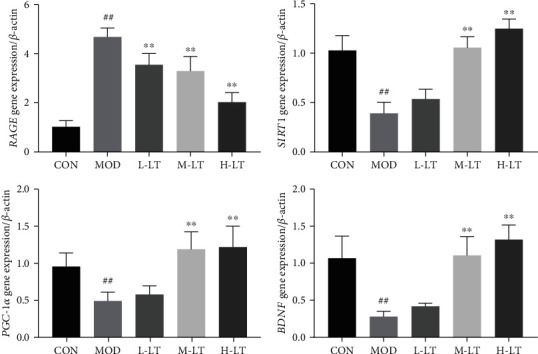
Expression levels of *RAGE*, *SIRT1*, *PGC-1α*, and *BDNF* mRNAs in rats' brain tissues of each group (*n* = 6). Compared with the CON group: ^#^*P* < 0.05, ^##^*P* < 0.01; compared with the MOD group: ^∗^*P* < 0.05, ^∗∗^*P* < 0.01. L-LT: low-dose (100 mg/kg) l-theanine group; M-LT: medium-dose (200 mg/kg) l-theanine group; H-LT: high-dose (400 mg/kg) l-theanine group.

As shown in Figure 6, compared with the CON group, *RAGE* mRNA levels in the brain tissues of the MOD group were significantly increased, whereas those of *PGC-1α* and *BDNF* mRNA significantly decreased (*P* < 0.01). Compared with the MOD group, *RAGE* mRNA levels in the brain tissues of the l-theanine-treated groups significantly decreased (*P* < 0.01). In contrast, the levels of *SIRT1*, *PGC-1α*, and *BDNF* mRNAs were significantly increased in the M-LT and H-LT groups (*P* < 0.01).

### 3.7. Effects of l-Theanine on the Expression of Key Proteins in the Brain Tissues of Experimental Rats

**Figure 7:**
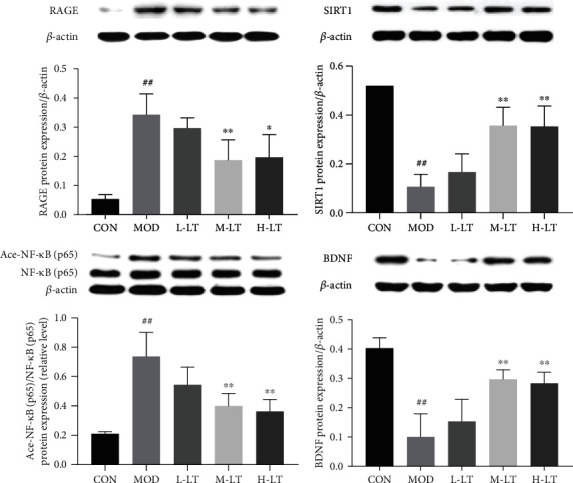
Protein expression of RAGE, SIRT1, Ace-NF-*κ*B (p65), and BDNF in rats' brain tissues of each group (*n* = 3). Compared with the CON group: ^#^*P* < 0.05, ^##^*P* < 0.01; compared with the MOD group: ^∗^*P* < 0.05, ^∗∗^*P* < 0.01. L-LT: low-dose (100 mg/kg) l-theanine group; M-LT: medium-dose (200 mg/kg) l-theanine group; H-LT: high-dose (400 mg/kg) l-theanine group.

As shown in Figure 7, the protein expression levels of RAGE, SIRT1, Ace-NF-*κ*B (p65), and BDNF in the brain tissues of the MOD group were significantly altered in comparison with the CON group (*P* < 0.01). Significant changes in those protein expression levels were observed in the M-LT and H-LT groups (*P* < 0.05 or *P* < 0.01), whereas no significant change was observed in brain tissues (*P* > 0.05).

**Figure 8:**
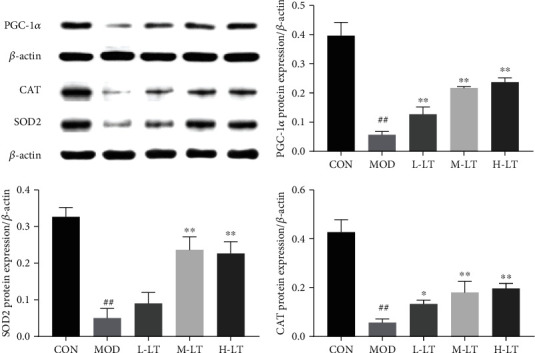
Protein expression of PGC-1*α*, SOD2, and CAT in rats' brain tissues of each group (*n* = 3). Compared with the CON group: ^#^*P* < 0.05, ^##^*P* < 0.01; compared with the MOD group: ^∗^*P* < 0.05, ^∗∗^*P* < 0.01. L-LT: low-dose (100 mg/kg) l-theanine group; M-LT: medium-dose (200 mg/kg) l-theanine group; H-LT: high-dose (400 mg/kg) l-theanine group.

As shown in Figure 8, the expression levels of PGC-1*α*, SOD2, and CAT proteins in the brain tissues of rats in the MOD group were significantly lower than those of the CON group (*P* < 0.01). Compared with the MOD group, the protein expression levels of SOD2 in the brain tissues of rats in the L-LT group did not significantly change (*P* > 0.05), whereas those of PGC-1*α* and CAT significantly increased (*P* < 0.05). The expression levels of PGC-1*α*, SOD2, and CAT proteins in the brain tissues of rats in the M-LT and H-LT groups were significantly increased (*P* < 0.01).

**Figure 9:**
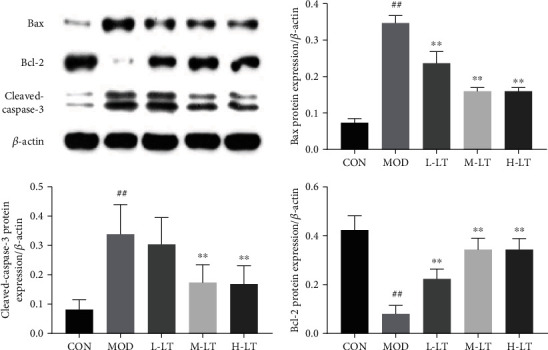
Protein expression of Bcl-2, Bax, and cleaved-caspase-3 in rats' brain tissues of each group (*n* = 3). Compared with the CON group: ^#^*P* < 0.05, ^##^*P* < 0.01; compared with the MOD group: ^∗^*P* < 0.05, ^∗∗^*P* < 0.01. L-LT: low-dose (100 mg/kg) l-theanine group; M-LT: medium-dose (200 mg/kg) l-theanine group; H-LT: high-dose (400 mg/kg) l-theanine group.

As shown in Figure 9, compared with the CON group, the significant changes in protein expression levels of Bcl-2, Bax, and cleaved-caspase-3 were observed in the brain tissues of rats in the MOD group (*P* < 0.01). Compared with the MOD group, the protein expression levels of cleaved-caspase-3 in the brain tissues of rats in the L-LT group did not significantly change (*P* > 0.05), whereas the protein expression levels of Bcl-2 and Bax were significantly changed (*P* < 0.01). Additionally, significant changes in the protein expression levels of Bcl-2, Bax, and cleaved-caspase-3 were observed in the brain tissues of rats in the M-LT and H-LT groups (*P* < 0.01).

## 4. Discussion

A*β* is a transmembrane protein that abnormally accumulates in the brains of patients with AD and can penetrate the cell membrane of neurons, alter the cell osmotic balance, induce neurotoxicity, and lead to memory impairment and neuronic loss [29]. Notably, AGE concentration positively correlates with the degree of pathological changes in patients with cognitive impairment [3, 30]. Long-term exposure to a high concentration of d-galactose significantly increases AGEs and A*β*_1-42_ levels in the brain of mice and results in cognitive impairment [31, 32]. Reducing AGEs and A*β*_1-42_ levels in the brain is essential for the prevention of cognitive impairment. A*β*_1-42_ concentration in the cerebral cortex and hippocampus was reportedly significantly decreased in mice fed l-theanine for 5 weeks [33]. We found that the concentrations of AGEs and A*β*_1-42_ were significantly increased when induced with d-galactose and then decreased after l-theanine treatment, which is consistent with the fact that nutritional intervention with l-theanine effectively reduced the amount of AGEs and A*β*_1-42_ in brain tissues [33, 34].

Progressive cognitive impairment is also related to dynamic changes in ACh and AChE activity [35]. In this study, when induced by d-galactose, the activity of AChE was significantly increased in rat brains. l-Theanine can inhibit ACh degradation by decreasing the activity of AChE in brain damage induced by scopolamine (1 mg/kg, i.p.) [36]. Similarly, our results showed that l-theanine inhibited the AChE activity and restored normal levels of ACh in brain tissues, thereby repressing the damage caused by AGEs and A*β*_1-42_. These findings were consistent with the fact that l-theanine blocked the increase in A*β*_1-42_ content and AChE activity induced by external stress and prevented AD [33, 34, 36].

AGEs and A*β*_1-42_ can bind to RAGE and promote intracellular oxidative stress production, with the activation of the RAGE signal pathway [8, 37]. The brain is the most vulnerable organ to oxidative stress due to its high metabolic activity, high lipid content, and limited antioxidant defense capacity [13]. According to the theory of free radical aging, maintaining the balance of oxidative stress contributes to preventing or delaying age-related diseases [38]. Previous studies have shown that d-galactose induces oxidative stress damage and mitochondrial dysfunction and promotes the production of AGEs [13, 39]. In this study, we found that the gene and protein expression levels of RAGE were increased in the brains of d-galactose-induced glycosylated model rats, and significant changes were observed in various oxidative stress indexes. Mitochondria are the primary energy production site for normal cell physiological activities and enhance brain cognitive ability [40]. However, excessive production of active oxidative stress products leads to mitochondrial damage and dysfunction, a key factor in the occurrence of neurodegenerative diseases, such as AD [41]. Studies have shown that PGC-1*α* is a key regulator of mitochondrial biosynthesis and function [40, 42], whereas antioxidant enzymes such as SOD2 and CAT protect the mitochondria from oxidative stress, exerting neuroprotective effects [43]. l-Theanine increased SOD2 and CAT protein levels in the brains of rats exposed to d-galactose, thereby alleviating the imbalances in oxidative stress components, inhibiting the production of AGEs, and preventing AGE-induced neurotoxicity in brain tissues. l-Theanine also upregulated PGC-1*α* mRNA and protein in the brains of d-galactose-treated rats, contributing to normal biosynthesis of mitochondria in rat brain tissues and positively regulating oxidative metabolism. In addition, l-theanine recalibrated the redox balance in the mitochondria to resist the occurrence and development of neurodegenerative diseases [44]. This may be attributed to the antioxidant activity of l-theanine, as reported by Ben and Jo; indeed, l-theanine maintains the normal biological function of mitochondria, thereby reducing neurotoxicity to protect nerve cells [45, 46].

Chronic inflammation, which also high related with AD, is a pathological condition characterized by a continuous active inflammatory response and tissue destruction [47]. Persistent oxidative stress injury is also a signal of chronic inflammation and is closely associated with the pathological and physiological changes of many age-related degenerative diseases [48]. Inflammation can mitigate abnormalities and promote tissue healing in acute cases at low levels. However, a long-term high level of inflammation could seriously damage host tissues [49]. The transcription factor NF-*κ*B is a key factor regulating inflammation. Of note, NF-*κ*B acetylation activates its regulatory ability. Continuous increases in Ace-NF-*κ*B (p65) protein levels promote the secretion of downstream proinflammatory factors, such as IL-1*β*, IL-6, and TNF-*α*, and induce systemic inflammation [50]. In addition, activated NF-*κ*B (p65) positively correlates with high expression of RAGE [51]. Activated NF-*κ*B, RAGE, and proinflammatory factors form a vicious circle, causing a state of persistent pathological inflammation in the brain. As an effective immunomodulator [24, 52, 53], l-theanine was found to inhibit the activation of NF-*κ*B (p65) and the expression of proinflammatory factors, such as TNF-*α*, IL-1*β*, and IL-6 in the rat brain, thereby promoting resistance to nerve cell damage. Accordingly, l-theanine inhibited Ace-NF-*κ*B protein expression in rat brains exposed to d-galactose and downregulated RAGE mRNA and protein expression, thus preventing an increase in proinflammatory markers, such as IL-1*β*, nNOS, TNF-*α*, and IL-6. These findings suggest that l-theanine could be used to regulate the persistent pathological inflammatory state in the brain tissues of d-galactose-treated model rats and restore inflammatory balance in brain tissues.

Apoptosis is an active form of programmed cell death, which is closely related to AD lesions and is regulated by specific proteins, including Bcl proteins and caspases [54]. Cho and Di reported that l-theanine has antineuronal apoptosis effects, suggesting applications in the prevention and treatment of neurodegenerative diseases [34, 55]. l-Theanine inhibited the expression of the apoptotic proteins, including Bax and cleaved-caspase-3. Furthermore, l-theanine promoted the expression of the antiapoptotic protein Bcl-2, which controls the level of neuronic apoptosis in brain tissues and maintains brain environment stability. Additionally, H&E staining of hippocampal rat tissues confirmed that l-theanine blocked d-galactose-induced neuronal apoptosis.

Impairment of neuronal plasticity due to normal aging is paralleled by neuronal damage, apoptosis, and reduced cognitive ability [17]. Neurotrophins and their receptors, particularly BDNF, are expressed in highly malleable brain regions (e.g., the hippocampus and cerebral cortex), which are considered molecular mediators of functional and morphological synaptic plasticity. These molecules are also essential for neuronal proliferation, excitability, synaptic transmission, and plasticity [56, 57]. In addition, BDNF plays a vital role in supporting the survival and growth of sensory and motor neurons, all of which are vital factors for learning and memory [58]. Moreover, some exogenous substances enhance BDNF expression, thereby inhibiting neuronal apoptosis and reducing neurodegenerative changes [59, 60]. We found that d-galactose inhibited the expression of BDNF, whereas l-theanine enhanced the expression of BDNF mRNA and protein in the brains of d-galactose-treated rats, thereby preventing d-galactose-induced hippocampal neuron dysfunction. These findings are consistent with a report that l-theanine increases BDNF content in the serum or hippocampal tissue and inhibits the effects of neurotoxicity caused by exogenous stress on neurodegenerative diseases [55]. Furthermore, it has been reported that PGC-1*α* overexpression can reverse the inhibition of BDNF mRNA expression in neurons of APP/PS1 transgenic mice [61]. More importantly, the increased expression of upstream PGC-1*α* can upregulate the BDNF expression and counteract the effect of A*β*_1-42_ on neuronal apoptosis [61, 62]. Collectively, these findings indicate that PGC-1*α* plays a pivotal role in BDNF upregulation. Our results suggest that l-theanine may also upregulate the level of PGC-1*α* protein in rat hippocampus exposed to d-galactose, recover the level of BDNF, and improve the d-galactose-induced brain damage.

Homeostasis of the internal environment is essential for promoting healthy aging of the body [28]. SIRT1 is a highly conserved protein that functions in deacetylation and maintenance of the homeostasis of the internal environment [28, 63]. SIRT1 is involved in several biological processes, such as oxidative stress, inflammatory reactions, apoptosis and senescence, mitochondrial biosynthesis, glucose and lipid metabolism, and oxidative metabolism mediated by downstream RAGE factors, such as SOD2, CAT, NF-*κ*B, Bax, and PGC-1*α* [63, 64]. Moreover, SIRT1 plays important roles in inhibiting the occurrence and development of neurodegenerative diseases, protecting nerve cells, and maintaining normal nerve function [14]. Abnormal increases in AGEs and A*β*_1-42_ induced by d-galactose can mediate inflammation, oxidative stress injury, mitochondrial dysfunction, and apoptosis and decrease BDNF and ACh levels, resulting in induction of the pathological processes of neurodegenerative diseases [13, 65, 66]. Therefore, reducing the concentrations of AGEs and A*β*_1-42_ and the RAGE expression in brain tissue is critical for preventing and treating neurodegenerative diseases. In our study, we showed that l-theanine inhibited the accumulation of AGEs and A*β*_1-42_ in the brains of model rats exposed to d-galactose and facilitate the maintenance of normal BDNF and ACh levels. Additionally, l-theanine blocked NF-*κ*B acetylation and downregulated RAGE mRNA and protein levels by activating SIRT1 [67]. Furthermore, l-theanine maintained the balance of inflammatory responses, modulated redox balance and mitochondrial biosynthesis, and reduced neuronal apoptosis in the hippocampus, thereby facilitating homeostasis of brain tissue and alleviating brain injury induced by d-galactose.

Prevention and treatment of d-galactose-induced brain injury by l-theanine could apply to other tissues besides the brain. For example, previous studies have shown that age-related degenerative diseases can be controlled or attenuated by activating the energy metabolite adenylate-activated protein kinase (AMPK) [68]. The longevity protein SIRT1 is required for AMPK activity [69]. We have previously shown that l-theanine has protective effects in the livers of d-galactose-treated rats and promotes glucose metabolism through the AMPK signaling pathway, resulting in reduced sugar concentrations in the body [26, 27]. Therefore, we speculate that l-theanine can inhibit the positive reaction of Schiff base formation between d-galactose and free amino groups, along with its reduction of AGEs and A*β*_1-42_ concentrations, thereby alleviating d-galactose-induced damage in rats' brain tissues.

**Figure 10:**
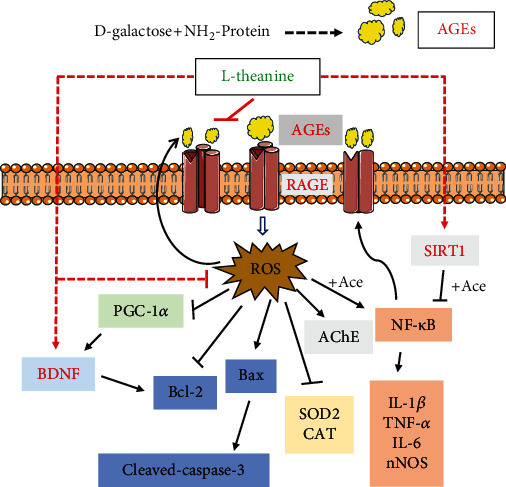
Potential alleviation mechanisms of l-theanine on brain injury in d-galactose-induced glycosylation model rats.

Thus, the potential alleviation mechanism of l-theanine on brain injury induced by d-galactose is presented in Figure 10. Treatment with l-theanine decreased A*β*_1-42_ and mitigated AGEs/RAGE-induced brain damage by upregulating SIRT1 and BDNF proteins.

## 5. Conclusion

l-Theanine was found to mitigate brain damage via inhibiting AGEs/RAGE signaling pathways and upregulating SIRT1 and BDNF, which indicate that l-theanine may be a potential functional food to prevent AD and promote healthy aging. For future work, studies of further mechanisms of l-theanine on different tissues of rats will be required. It would also be desirable to examine the metabolism of l-theanine in a rat model of d-galactose-induced brain damage.

## Figures and Tables

**Table 1:** Primers used for qPCR.

Gene	Forward and reverse primer (5′–3′)	Product size (bp)
*β*-Actin	F: ACATCCGTAAAGACCTCTATGCC R: TACTCCTGCTTGCTGATCCAC	223
*RAGE*	F: CTGCCTCTGAACTCACAGCCAATG R: GTGCCTCCTGGTCTCCTCCTTC	155
*SIRT1*	F: AAAGGAAATATATCCCGGACA R: TTTGGATTCCTGCAACCTG	134
*PGC-1α*	F: AATCAAGCCACTACAGACACC R: TCTCTGCGGTATTCGTCCCTC	148
*BDNF*	F: GTCCCGGTATCAAAAGGCCAAC R: AGTGCCTTTTGTCTATGCCCCT	103

## Data Availability

The data used to support the findings of this study are included within the article.

## Abbreviations

A*β*: *β-*Amyloid
ACh: Acetylcholine
AChE: Acetylcholinesterase
AD: Alzheimer's disease
AGEs: Advanced glycation end products
AMPK: AMP-activated protein kinase
BDNF: Brain-derived neurotrophic factor
CAT: Catalase
CON: Control group
GSH: Glutathione peroxidase
H-LT: High-dose l-theanine group
IL: Interleukin
L-LT: Low-dose l-theanine group
MDA: Malondialdehyde
M-LT: Medium-dose l-theanine group
MOD: Model group
NF-*κ*B (p65), nNOS: Neuronal nitric oxide synthase
NOS: Nitric oxide synthase
PGC-1*α*: Peroxisome proliferator-activated receptor-*γ* coactivator-1*α*
RAGE: Receptor for AGEs
ROS: Reactive oxygen species
SD: Sprague-Dawley
SIRT1: Sirtuin1
SOD: Superoxide dismutase
T-AOC: Total antioxidant capacity
TNF-*α*: Tumor necrosis factor-*α*.
